# Effects of β-Fructans Fiber on Bowel Function: A Systematic Review and Meta-Analysis

**DOI:** 10.3390/nu11010091

**Published:** 2019-01-04

**Authors:** Jan de Vries, Cindy Le Bourgot, Wim Calame, Frédérique Respondek

**Affiliations:** 1 De Vries Nutrition Solutions Inc., 7213 CE Gorssel, The Netherlands; nutritionsolutions@me.com; 2 R & D Department, Tereos, Rue de Senlis, 77230 Moussy-Le-Vieux, France; frederique.respondek@wanadoo.fr; 3 StatistiCal BV, 2241 MN Wassenaar, The Netherlands; w.calame@kpnplanet.nl

**Keywords:** bowel function, constipation, dietary fibers, fructooligosaccharides, prebiotic

## Abstract

The aim of this systematic review and meta-analysis was to assess the effects of β-fructan supplementation on bowel function in healthy volunteers and patients. The search process was based on the selection of publications listed in the Pubmed and EUPMC database until December 2017, plus two unpublished studies, to identify studies evaluating the impact of β-fructans on bowel movement and stool parameters. Forty-seven publications were selected for inclusion. Primary parameter was frequency of bowel movements, evaluated by the number of defecations per day during the study period. Secondary outcomes were stool consistency, stool dry and wet weights, and transit time. Short-chain (DP < 10) β-fructans contributed to increased stool frequency (0.36 defecation +/− 0.06 per day; *p* < 0.001), while no significant effect was reported with long-chain (DP ≥ 10) β-fructans (−0.03 +/− 0.11, *p* = 0.82). A minimal increase in stool wet weight was also statistically demonstrated with short-chain β-fructans. Moreover, the meta-analysis highlighted significant differences in stool consistency in contrast to fecal dry weight after β-fructan supplementation. This systematic review and meta-analysis indicates that short-chain β-fructan supplementation has a positive effect on bowel function by significantly increasing the frequency of bowel movements.

## 1. Introduction

Functional constipation is a gastrointestinal syndrome based on at least two of the following symptoms during the last three months in at least 25% of defecations: (a) straining; (b) lumpy or hard stools; (c) sensation of incomplete evacuation; (d) sensation of anorectal blockage; (e) manual maneuvers to facilitate; and, (f) as well as fewer than three bowel movements per week [1]. The median prevalence of functional constipation in adults is around 16% globally and varies from 0.7 to 79% depending on the definition used in different regions [2,3,4]. This disorder is more common in women than in men and found to increase with age [5,6]. Moreover, it is one of the main reasons to visit a gastroenterologist [2,7]. Inadequate dietary fiber intake and inadequate fluid consumption are some of the multiple etiologies of constipation and gastrointestinal discomfort [8]. 

Dietary fiber is made up of carbohydrate polymers with three or more monomeric units that are neither digested nor absorbed in the human intestine [9]. Adequate fiber intake is broadly recognized around 25 g per day for normal laxation [10,11], whereas average daily consumption is generally lower (by 5–10 g/day) than recommended in developed countries [12]. Poor sensory attributes of foods enriched in fibers are sometimes limiting their consumption [13,14]. Treatment of functional constipation with dietary and lifestyle adjustments, including the higher consumption of dietary fibers, is usually recommended prior to utilization of osmotic or stimulant laxatives [8]. It is also the most common action spontaneously used for managing constipation in self-reported cases [15]. Despite the fact that dietary fibers in general are well known for their beneficial effects on bowel function, such as improving regularity and increasing fecal weight, the physiological effect may depend on the type of fiber. In fact, dietary fibers exhibit a diverse range of physiochemical properties which are important determinants of their effects on gastrointestinal function. It is broadly acknowledged that insoluble fibers were linked with laxation benefits, as they adsorb water in the gastrointestinal lumen and lead to larger and softer fecal mass [8,16,17]. However, scientific evidence supports also the efficacy of soluble fibers on bowel function.

β-fructans are an example of soluble dietary fibers [18]. They are mostly linear, characterized by a β1-2 linkage between molecule of fructose, varying in degree of polymerization (DP) from two to about 200 in plants, and potentially glucose at the terminal position [19]. They are naturally present in various fruits and vegetables (including chicory). Linear long-chain fructans are mostly extracted from chicory roots. Oligomeric fructans (DP 3–9), usually called oligofructose or fructo-oligosaccharides, are mostly obtained from the hydrolysis of inulin or enzymatic synthesis from sucrose (beet or cane) [19]. They are selectively fermented by a limited number of bacteria in the large intestine, especially Bifidobacteria, which are rather sensitive to their degree of polymerization [20,21]. The potential of β-fructans to provide health benefits in humans is known for several years [22]. Their efficacy on bowel function has been studied in humans and acknowledged by several health authorities [23,24]. However, it has not been clearly systematically reviewed for all combined β-fructans, including short and long-chain molecules, neither for short-chain β-fructans only.

Therefore, this paper aims to systematically review the effects of all β-fructans on bowel function in humans and more particularly on the frequency of bowel movements, stool consistency, fecal dry and wet weight, and transit time. To address potential quantitative effects of β-fructans on the aforementioned stool parameters a meta-regression was conducted, including the impact of the degree of polymerization, dose, interval of study, age, and body mass index (BMI).

## 2. Materials and Methods

We conducted a systematic review and a meta-analysis to evaluate the effect of β-fructans on bowel function. Primary outcome was the frequency of bowel movements, as evaluated by the number of defecations per day during the study period. Secondary outcomes were stool consistency, stool dry and wet weights, and transit time. The present review was conducted in accordance with the Preferred Reporting Items for Systematic Reviews and Meta-Analyses (PRISMA) guidelines [25].

### 2.1. Formulation

The primary question was “Does regular intake of β-fructans increase frequency of bowel movements and moreover, is there a different effect according to DP of β-fructans (short-chain DP < 10 vs. long-chain DP ≥ 10)?”.

The secondary question was “Does regular intake of β-fructans impact other bowel functions as stool consistency, stool wet and dry weight, and transit time?”.

### 2.2. Eligibility Criteria

Interventions were considered eligible for the systematic review if the following criteria were met: (1) the study was conducted in a human population aged > 3 years; (2) β-fructans were tested as single supplementary ingredient applied in a product or dietary supplementation; (3) a relevant outcome measurement of bowel function, including frequency of bowel movements, stool consistency, as measured with the Bristol Stool Scale, stool wet or dry weight, and transit time was examined; and, (4) the publication was written in the English language. The meta-analysis was conducted only on those studies that included a placebo in the study design.

### 2.3. Literature Search and Study Selection

A comprehensive literature search using PubMed and EUPMC was performed to identify intervention studies in human populations through 20 December 2017 (to be considered as the end date of the digital search process). The full search string used in each database is: (β-fructan* OR fructan OR fructooligosaccharide* OR fructo-oligosaccharide* OR oligofructose OR FOS OR inulin OR Neosugar) AND (stool OR fecal OR faecal) AND (frequency OR bowel movement OR bowel habit OR defaecation OR regularity OR consistency OR constipation OR volume OR output OR weight OR laxati* OR transit time) NOT animal*.

Supplementary literature searches involved examining the reference lists of all relevant studies and pertinent reviews to identify articles that were not captured in the initial search. Two unpublished study reports, funded by Tereos and matching eligibility criteria, were also included in the final database.

**Figure 1:**
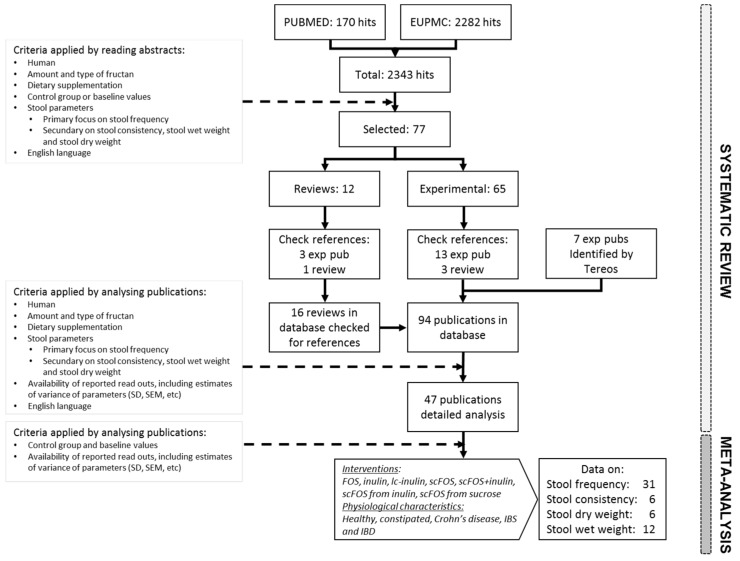
Flow diagram showing the results of the systematic literature search and meta-analysis on stool parameters.

The search flow is illustrated in Figure 1. Two independent reviewers (JdV and CLB) screened the titles and abstracts for relevance to the systematic review and meta-analysis. Potentially eligible articles were reviewed jointly to resolve any discrepancies regarding study selection. 

### 2.4. Data Extraction

The following general study information was extracted using FileMaker Pro software by JdV: first author; journal; year; volume; page; discussion issue; physiological characteristics; age; BMI; BW; number; number of females; number of males; type of intervention; type of β-fructan, including average and range of DP if provided; dose of β-fructan; type of administration; measured outcome parameter; method used to measure outcome parameter; units; day of measurement; mean value; and, SD value.

Outcome data for frequency of bowel movements (number/day), stool consistency (score based on Bristol Stool Scale [26]), stool dry and wet weights (g/day), and transit time (h) were extracted. Where bowel movements were reported as number/week a recalculation to number/day was conducted. Extracted data included baseline and trial end values, change-from-baseline values, statistical significance of change values, and differences in the trial end value between the intervention and control arm when it was available in the publication.

### 2.5. Risk of Bias in Individual Studies and Across Studies

Publication bias was assessed with both the Begg’s test as well as the Egger test applying regression symmetry [27,28].

### 2.6. Statistical Analysis

A meta-analysis on stool frequency included studies reporting on placebo-controlled interventions longer than one-day. Per study the effect of intervention at the end was applied in the meta-analysis. There were no differentiations in dose of the β-fructan neither in the physiological characteristics of the volunteers (included values of healthy individuals, constipated individuals, IBS, IBD, and Crohn’s disease patients). Subsequently an analysis on short-chain (DP < 10) and long-chain (DP ≥ 10) β-fructans was made. Accordingly, a meta-regression on dose, DP, duration of the study, BMI, and age was conducted, and a separate analysis for short-chain β-fructans and long-chain β-fructans, but then without DP as independent factor. Additionally, the following parameters were analyzed when possible (enough data): stool consistency, stool dry weight, stool wet weight and transit time. The meta-analysis of the outcome in the various studies was performed using random-effects models using Cohen’s method to compute the standardized mean difference. The significance of the overall effect was calculated by a *z*-score being the ratio of the overall effect to its standard error, after which comparison with the standard normal distribution was done. Heterogeneity was assessed via the *I*^2^ statistic as well as Cochran’s Q [29]. Meta-regression (meta-analysis regression) was performed to analyze the association between heterogeneity in the outcome of the meta-analysis and one or more characteristics of the studies [30], followed by a Monte Carlo permutation test for meta-regression [31], applying at least 5000 runs. Finally, to evaluate a potential dosing effect by the consumption of fructans on the effect size with respect to stool frequency has been addressed by a sigmoidal (Pseudo-Hill) equation thatis based on the difference in the mean values between treatment and placebo per study. The model used: effect size = minimum + (maximum − minimum) × (1 − e^−kx^), with k as rate constant.

The software used was STATA version 12.1 (StataCorp, College Station, TX, USA). A *p*-value below 0.05 was considered to detect statistical significance applying two-sided testing. 

## 3. Results

### 3.1. Study Selection

The search yielded 170 references in PUBMED and 2282 references in EUPMC, of which 77 articles were retained for full-text screening and reference list review. The 77 articles included both original experimental research publications (*n* = 65) and reviews (*n* = 12). Screening of reference lists from all relevant review articles and eligible experimental studies resulted in 23 additional original publications, for a total of 88 experimental publications in the database. At the end, 45 publications were eligible for inclusion, to which were added two unpublished human intervention trials that were registered under number NCT01847950 and NCT03707002.

### 3.2. Study Characteristics

The characteristics of all 47 publications are presented in Table 1. The studies were conducted between 1995 and 2017. Among all publications that were included in the systematic review, 41 of the studies were conducted as placebo-controlled trials, with 20 of cross-over and 21 of parallel trials. The six remaining publications in review were five sequential trials and one single arm intervention trial.

Most of the studies were conducted in healthy subjects (*n* = 29). Other studies were performed in constipated people (*n* = 9), subjects with IBS, IBD, or Crohn’s disease (*n* = 4), and people with other specific health status (*n* = 5). 

Publications were divided into different categories according to the DP of β-fructans that were tested (short-chain β-fructans and long-chain β-fructans). 

Specific information on the chain length of the used β-fructans was provided in or could be retrieved from 44 studies that were included in the systematic review. When a study used a mixture of β-fructans with short and long-chains, we decided that the majority of the β-fructan mixture (>50%) goes to corresponding category, e.g. if a mixture contains 60% of β-fructan with DP<10, this study was added to the short-chain β-fructans category. Some publications provide insufficient details for an adequate description of the β-fructan used, particularly on this average DP. Eighteen of the studies have been conducted using long-chain β-fructans or mixtures of β-fructans as compared with 28 using short-chain β-fructans. The major part of the trials has a duration of treatment ≥ 7 days up to 27 days (n = 23 studies) compared, with three studies with a duration < 7 days and 21 studies ≥ 28 days of β-fructan supplementation. The range of supplementation was 1.3 to 30 g β-fructan per day with a mean value of 12 g/day.

### 3.3. Meta-Analysis

For the meta-analysis, only placebo-controlled studies were used, both in cross-over and with a parallel design protocol in healthy, constipated, IBS, IBD, and Crohn’s disease individuals. At the end, the meta-analysis was performed on 31 observations for frequency of bowel movements, 18 observations for stool consistency, of which six measured with the generally accepted Bristol Stool Scale, six observations for stool dry weight, and 12 observations for stool wet weight. Unfortunately, only a limited number of studies reported data on transit time, therefore we could not include this variable in the meta-analysis.

#### 3.3.1. Primary Outcome: Stool Frequency

A meta-analysis was performed on 29 experimental research studies, representing 31 observations in total for stool frequency, due to more than one experiment within some publications, such as more than one dose tested, or more than one β-fructan used (Table S1).

**Figure 2:**
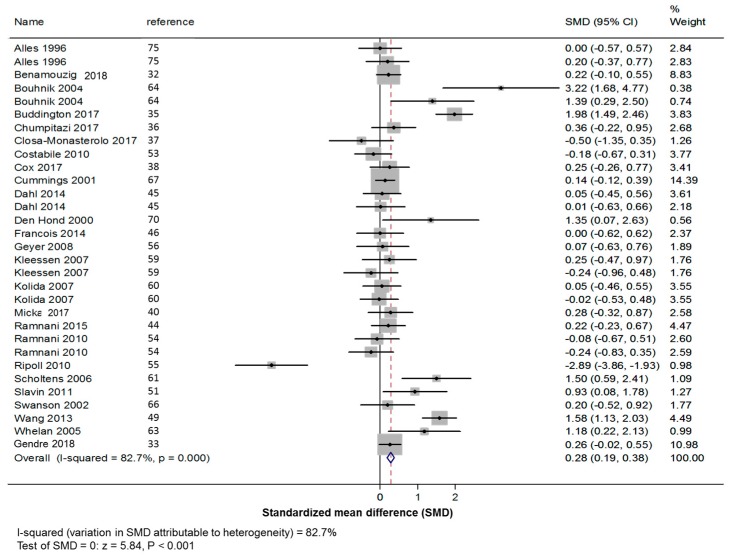
Forest plot for the effect of all β-fructans on stool frequency. SMD: standardized mean difference (number of bowel movements/day); CI: confidence interval.

All β-fructans contributed to increase frequency of bowel movements (+0.28 +/− 0.06 defecation per day; *z*: 5.84, *p* < 0.001; Figure 2). 

**Figure 3:**
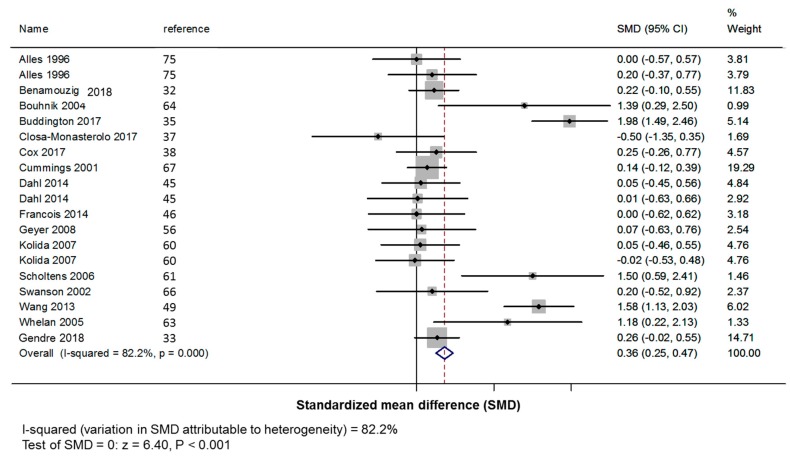
Forest plot for the effect of short-chain β-fructans (DP < 10) on stool frequency. SMD: standardized mean difference (number of bowel movements/day); CI: confidence interval.

**Figure 4:**
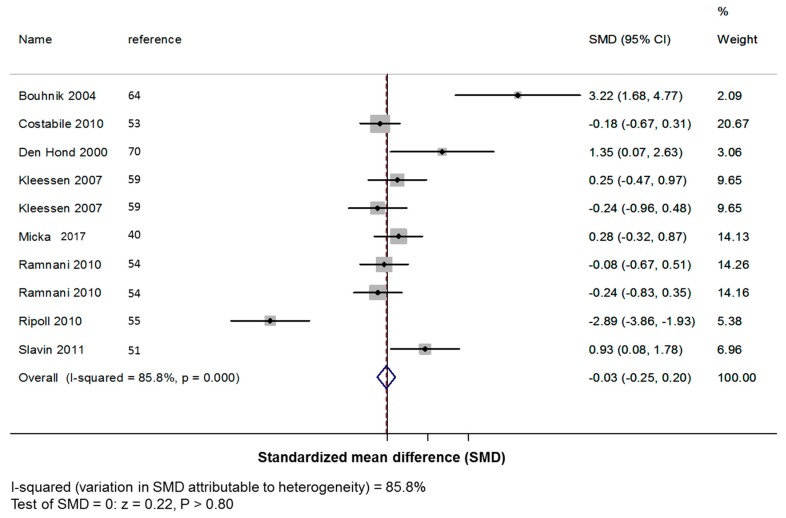
Forest plot for the effect of long-chain β-fructans (DP ≥ 10) on stool frequency. SMD: standardized mean difference (number of bowel movements/day); CI: confidence interval.

This increase of bowel movement frequency was mostly explained by short-chain β-fructans (+0.36 +/− 0.06 defecation per day; *z*: 6.40, *p* < 0.001; Figure 3) than long-chain β-fructans (−0.03 +/− 0.11 defecation per day; *z*: 0.22, *p* = 0.805; Figure 4). 

**Figure 5:**
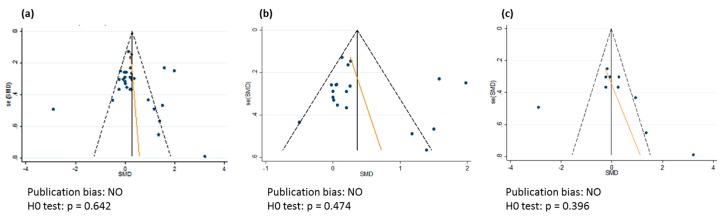
Funnel plot of publication bias for the effect of β-fructans on stool frequency. (**a**) Data for all β-fructans; (**b**) Data for short-chain β-fructans (DP < 10); and, (**c**) Data for long-chain β-fructans (DP ≥ 10).

All analyses showed heterogeneity of more than 80% (*p* < 0.001), and publication bias was not encountered (Figure 5).

#### 3.3.2. Secondary Outcomes: Stool Consistency, Fecal Dry and Wet Weights

All combined β-fructans did yield a significant positive impact on stool consistency when analysis was performed on studies applying the Bristol Stool Scale score (Table 2). This effect was even more outspoken with a DP value below 10. Unfortunately, only one study applied a DP value higher than 10, therefore no conclusion on the length of the polymer on this particular efficacy could be investigated. No effect was observed on stool dry weight. However, a significant increase in the stool wet weight was demonstrated with all β-fructans (*p* < 0.01), as well as with the short-chain β-fructans (*p* < 0.02), while no significant effect was reported with long-chain β-fructans (*p* = 0.26). In the latter, heterogeneity was not observed, neither was publication bias (all *p*-values > 0.25).

### 3.4. Meta-Regression on Frequency of Bowel Movements

Our systematic review provided enough observations on frequency of bowel movements to conduct a meta-regression. There were too little observations on the other bowel parameters for this. Various confounders have been used in the meta-regression, such as the dose applied in the study, the β-fructan degree of polymerization, the day of final measurement, the age, and the BMI of subjects (Table S1). The results showed that the frequency of bowel movements is significantly dependent on BMI (*p* = 0.023; after applying Monte Carlo with 10.000 runs the *p*-value increased to 0.03). As it turned out: the higher the BMI of the subject, the greater the impact of β-fructan on increasing the frequency of bowel movements.

**Figure 6:**
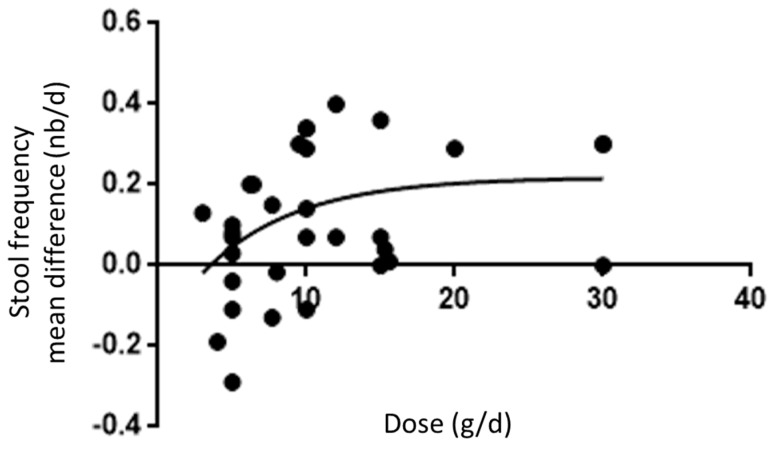
Dose-response curve for the effect of β-fructans on delta mean stool frequency. Sigmoidal relationship applying Pseudo-Hill modelling between the dose of β-fructans applied and the difference in outcome in stool frequency between consumption of β-fructans versus that of placebo expressed as the difference in mean values (number of bowel movements/day). Each point represents one single study.

Figure 6 depicts the association between the dose of β-fructans consumed and the impact on the change in stool frequency. The model describes the relationship, as follows: delta mean = 0.17 − 0.43e^−0.21x^. The graph drawn shows that already at a dose of 10 grams per day 60% efficacy is reached, increasing the dose to 18 grams a day yields 90% efficacy.

## 4. Discussion

The aim of the current paper was to undertake a systematic review and a meta-analysis of human studies to evaluate the effect of β-fructan supplementation on bowel function. A focus was given on frequency of bowel movements; one of the outcomes recognized by the most up to date guidance from EFSA and US FDA to evaluate the beneficial physiological effect of dietary fibers on bowel function/laxation provided that the changes do not result in diarrhea [31,32]. Overall, the results showed that regular consumption of β-fructan significantly increases the frequency of bowel movements when compared to non-supplemented individuals in the general population with or without intestinal disorders. This effect is attributable to short-chain β-fructans, and not to long-chain β-fructans (DP ≥ 10). An increase of stool frequency (+0.36 defecations per day) by the regular consumption of short chain β-fructans is of importance, particularly in individuals with chronic constipation, a common symptom-based gastrointestinal disorder, to return to a more normal frequency of bowel movements (i.e more than three per week, less than three per day) [1]. BMI is a confounding parameter, with highest BMI inducing higher efficacy of β-fructans.

Contrary to linear dose-effect relationship that has been reported with cereal fibers [16], the effect of β-fructans seems to increase according to the dose until approximately 18 g/day with no substantial additional benefit to further increase the dose. Interestingly, it was previously shown that the well-known stimulation of Bifidobacteria observed with short-chain β-fructans is dose-related between 2.5 and 10 g/day, and above this dose, further increase becomes marginal [34]. 

This increase in stool frequency is the common range of what is reported in literature for other types of fibers or molecules. In a systematic review that was conducted by de Vries et al., the mean value of the increase in stool frequency was 0.34 ± 0.23 bowel movements/day after the consumption of 13.6 ± 6.4 g/day dietary fibers derived from cereals. The effect was significant when expressed in Δ in times/day per g/day fiber (0.004 ± 0.002) *p* < 0.05 [16]. With resistant maltodextrin ingestion, the increase in stool frequency was lower than observed in the present meta-analysis with a mean value around +0.71 (*p* < 0.001) bowel movement per week, thus approximating +0.10/day [35]. In 2015, EFSA provided a Scientific Opinion related to the effect of 12 g/day of native chicory inulin (DP ≥ 9) on the maintenance of normal defecation by increasing stool frequency of about one additional stool per week versus placebo [23]. In our meta-analysis, the increase in bowel movement frequency is higher with short-chain β-fructans (+0.36 defecations/day) as compared with long-chain β-fructans (−0.03 defecations/day). In the latter, the difference with placebo was not significant. In view of this health claim on inulin (long chains molecules) validated by the regulatory authorities, we can assume that supplementing the diet with short-chain β-fructans could also represent an improved strategy to counteract insufficient dietary fibre intake with beneficial effect on the frequency of bowel movements. 

In the current meta-analysis, we demonstrated that regular consumption of β-fructans by human subjects significantly increases the frequency of bowel movement, stool consistency, and stool wet weight when compared to non-supplemented individuals, important parameters for normal laxation. Dietary fibers can increase luminal bulk as they are not digested in the upper part of the intestine. Depending on their nature, they can also retain water and also increase fecal bulk. They can stimulate the growth of the microbial mass and its production of short-chain fatty acids (SCFA) [36]. As β-fructans are completely soluble and fermentable fibers, one of the explanatory mechanisms of these effects is their prebiotic activity [22]. They are known to stimulate the growth and the fermentative activity of specific carbohydrate-degrading bacteria in the gut microbiota, resulting in a greater bacterial mass being generally confirmed by a higher nitrogen excretion [37,38]. This is accompanied by higher production of SCFA, H2, and CO2, and higher water content of digesta [38], but also a modulation of the quantity and quality of bile acids present in the colon [39,40]. All SCFA, H2, and deconjugated bile acids issued from microbial fermentation are recognized to increase gastrointestinal motility [2]. β-fructans are well-known to stimulate the growth of beneficial bacteria, such as *Bifidobacterium* and *Lactobacillus*, and they are also fermented by other SCFA-producing bacteria, such as *Bacteroides* and *Roseburia* [20]. Thus, β-fructans demonstrate specific and complete fermentation in the large intestine inducing increase of bacterial mass and increased production of SCFA, and by this way, can lead to an increase in fecal mass with limited impact on stool volume [22,79]. 

Interestingly, we observed a different effect on frequency of bowel movements between shorter- and longer-chains. We hypothesize that this is possibly related to slightly different modulation of the microbiota composition and its fermentation, as illustrated by various in-vitro studies. While long-chain fructans may exert a more prolonged prebiotic effect in time [41], short-chain fructans are more easily-fermentable molecules, especially by Bifidobacteria and Lactobacilli, even if the dependency of growth promotion is highly strain specific [20,21,42]. In some bacteria, a switch in gene expression ending with an excretion of extracellular enzyme is needed prior to the utilization of long-chain fructans. In vitro, this is noticed by a longer lag-time before bacterial growth with long-chain than with short-chain fructans [20]. This could explain why we observed that the positive effect of β-fructan on bowel functions (i.e. stool frequency and wet fecal weight) is more attributable to short-chain β-fructan and not to longer chains. The duration of the studies testing long chains fructans and included in the meta-analysis was also possibly too short (less than 28 days) to lead to significant changes in the gut microbiota and thus to improve bowel functions on the longer-chain β-fructans [43,44,45].

In addition to stool frequency, changes in other bowel function parameters were also quantitatively evaluated. β-fructans supplementation, and more particularly short chain β-fructans, significantly increases stool wet weight and significantly softens stool consistency, while no significant effect was reported for stool dry weight and not enough studies reported data on intestinal transit to perform a meta-analysis. It was reported elsewhere that stool frequency correlated poorly with all transit measurements in constipated adults [46,47]. Although being statistically significant, the increase of fecal wet weight by 0.24 g/day and the softening effect seem limited from a physiological point of view, especially in comparison to what is observed on stool volume with non-soluble, less fermentable fibers, like cereal fibers [16]. Due to the lack of data, we could not perform the meta-regression for these variables. The dose might be important to draw final conclusion, as, for example, studies with higher daily dose of β-fructan (i.e. more than 10 g/day) showed a significant increase of fecal volume [48] and softening effect [49]. It is already worthy to note that the consumption of β-fructans did not induce the production of smaller and harder stools that could be sometimes associated with a higher frequency of bowel movements [36]. 

### 4.1. Tolerance

An increase in the frequency of bowel movements is a beneficial physiological effect of dietary fibers, provided that it does not result in diarrhea [32]. No diarrhea induced by β-fructans was reported in any of the studies that we included in this review, and, in addition, no difference of supplementation compliance was recorded. Our analyses show that β-fructans increase the frequency of bowel movements potentially impacting stool consistency, as assessed with the validated Bristol Stool Scale. β-fructans are generally considered to be well tolerated up to 30 g per day [80,81] that is in the range of the hardly achieved recommended daily intake of dietary fibers for normal laxation [12]. The first symptoms to occur are excessive flatus then borborygmi and bloating, abdominal cramps and diarrhea may only be expected for dosages higher than 50 g per day [80]. Generally, no significant gastrointestinal symptoms are observed for a daily dose up to 10–12 g per day [40,64,67,82], even in subjects with previous gastrointestinal complaints or in hypersensitive IBS patients [34,83,84]. On the contrary, some of their symptoms were improved with the regular intake of β-fructans. Higher frequency of minimal and transient gastrointestinal symptoms may appear above 10–12 g per day in some individuals, especially for occasional intake [48].

### 4.2. Limitations

Only randomized and controlled trials were included in the present meta-analysis. Stool frequency varied considerably between studies. We decided to include studies that were conducted in constipated or individuals suffering from IBS, IBD and Crohn’s disease in this review to have enough data to analyze. The different physiological characteristics can explain part of the heterogeneity. Furthermore, stool frequency is a discontinuous variable that is most often analyzed as a continuous variable. Only in the reports of Kleessen et al. and Micka et al. has this been taken into account in the statistical analysis. Our approach, considering stool frequency as a continuous variable, may have influenced the outcome of the analysis. The meta-regression only demonstrated a significant influence of BMI on the effects of β-fructans on stool frequency. However, other factors, such as duration of the study, dose, and length of the chain of the β-fructans, can also contribute to heterogeneity in study results. Nevertheless, funnel plots demonstrated no publication bias on the included studies. 

## 5. Conclusions

In conclusion, regular bowel movements are an important factor affecting the quality of life and they could be achieved by consuming more dietary fiber. When the intake of dietary fiber is insufficient, the consumption of foods containing β-fructans and more particularly short-chain β-fructans with a DP below 10, is a practical strategy that contributes to a significant increase of frequency of bowel movements with additional softening of stool consistency, probably by increasing the wet weight. 

## Figures and Tables

**Table 1:** Characteristics of all publications in review.

References	Experimental Design ^1^	*N*	Participants	Intervention ^2^	Comparator	Dose (g/Day)	Duration (Day)	Stool Frequency	Stool Consistency	Fecal Dry Weight	Fecal Wet Weight	Included in Meta-Analysis
Benamouzig, 2018 [32]	Parallel	150	Constipated	DP < 10 scFOS	Maltodextrin	5	42	Yes	Yes	No	No	Yes
Gendre, 2018 [33]	Parallel	187	Constipated	DP < 10 scFOS	Maltodextrin	5	42	Yes	Yes	Yes	Yes	Yes
Azpiroz, 2017 [34]	Parallel	36	IBS ^a^	DP ≥ 10	Maltodextrin	8	28	No	No	No	No	No
Buddington, 2017 [35]	Parallel	97	Constipated	DP < 10 OF	Maltodextrin	15	84	Yes	No	No	No	Yes
Chumpitazi, 2018 [36]	Cross-over	23	IBS ^a^	DP ≥ 10	Maltodextrin	6.2	3	Yes	Yes	No	No	Yes
Closa-Monasterolo, 2017 [37]	Parallel	22	Constipated	DP < 10 OF	Maltodextrin	4	42	Yes	Yes	No	No	Yes
Cox, 2017 [38]	Cross-over	32	IBD ^b^	DP < 10 OF	Glucose	12	3	Yes	Yes	No	No	Yes
Jinno, 2017 [39]	Parallel	64	Pregnant	DP < 10 scFOS	Sucrose	8	105	Yes	Yes	No	No	No
Micka, 2017 [40]	Cross-over	44	Constipated	DP ≥ 10	Maltodextrin	12	28	Yes **	Yes	No	No	Yes
Clarke, 2016 [41]	Cross-over	30	Healthy	Mixture	Maltodextrin	15	28	No	No	No	No	No ^d^
Garcia-Perris, 2016 [42]	Parallel	38	Gynecological cancer	Mixture	Maltodextrin	12	28	Yes	Yes	No	No	No
Meksawan, 2016 [43]	Cross-over	9	Peritoneal dialysis	DP < 10 OF	Placebo	20	30	Yes	Yes	No	No	No
Ramnani, 2015 [44]	Cross-over	38	Healthy	No detail	Maltodextrin	4.7	21	Yes	Yes*	No	No	Yes
Dahl, 2014 [45]	Parallel	98	Healthy	DP < 10 OF	Placebo	15.6 & 15.3	56	Yes	No	No	No	Yes
François, 2014 [46]	Cross-over NB	20	Healthy	DP < 10 OF	Placebo	30	14	Yes	Yes	No	Yes	Yes
Majid, 2014 [47]	Parallel	22	Healthy with enteral nutrition	DP ≥ 10	Maltodextrin	7	7	Yes	Yes	No	No	No
Respondek, 2014 [48]	Cross-over	36	Healthy	DP < 10 scFOS	Dextrose	11	1	Yes	Yes	No	No	No
Wang, 2013 [49]	Parallel	100	Constipated	DP < 10 OF	Placebo	1.26	10	Yes	No	No	No	Yes
Benjamin, 2011 [50]	Parallel	103	Crohn’s disease ^c^	DP < 10 OF	Maltodextrin	15	28	No	No	No	No	No ^e^
Slavin, 2011 [51]	Cross-over	12	Healthy	DP ≥ 10	Placebo	20	21	Yes	Yes	No	Yes	Yes
Yen, 2010 [52]	Parallel	10	Constipated	DP < 10 scFOS	Placebo	10	28	Yes	No	Yes	No	Yes
Costabile, 2010 [53]	Cross-over	32	Healthy	DP ≥ 10	Maltodextrin	10	21	Yes	Yes	No	No	Yes
Ramnani, 2010 [54]	Parallel	66	Healthy	DP ≥ 10	Placebo	5	21	Yes	Yes	No	No	Yes
Ripoll, 2010 [55]	Parallel	35	Healthy	DP ≥ 10	Sucrose	5	28	Yes	Yes	No	No	Yes
Geyer, 2008 [56]	Cross-over	16	Healthy	DP < 10 OF	Placebo	6.4	14	Yes	Yes *	No	No	Yes
Bouhnik, 2007 [57]	Single arm	12	Healthy	DP < 10 scFOS	None	8	28	No	No	Yes	Yes	No
De Preter, 2007 [58]	Cross-over	20	Healthy	DP ≥ 10	Maltodextrin	20	28	No	No	Yes *	Yes *	Yes
Kleessen, 2007 [59]	Parallel	45	Healthy	DP ≥ 10	Placebo	15	21	Yes **	Yes **	No	No	Yes
Kolida, 2007 [60]	Sequential	90	Healthy	DP < 10 OF	Maltodextrin	5 & 8	14	Yes	No	No	No	No
Scholtens, 2006 [61]	Cross-over	12	Healthy	DP < 10 OF	Maltodextrin	30	14	Yes	Yes	Yes	Yes	Yes
Dahl, 2005 [62]	Cross-over	15	Dysphagia	DP ≥ 10	Placebo	12.6	21	Yes	No	No	No	No
Whelan, 2005 [63]	Cross-over	10	Healthy	DP < 10 OF	Placebo	9.5	14	Yes	No	No	Yes	Yes
Bouhnik, 2004 [64]	Parallel	24	Healthy	DP < 10 scFOS DP ≥ 10	50:50 Malto/sucrose	10	7	Yes	Yes	No	No	Yes
Grasten, 2003 [65]	Parallel	14	Healthy	DP ≥ 10	Arabinoxylan-OS	13.3	21	Yes *	Yes *	No	No	No
Swanson, 2002 [66]	Parallel	68	Healthy	DP < 10 scFOS	Sucrose	3	28	Yes	Yes *	Yes *	No	Yes
Cummings, 2001 [67]	Parallel	244	Healthy	DP < 10 OF	Maltodextrin	10	28	Yes	Yes	No	No	Yes
Tahiri, 2001 [68]	Cross-over	11	Healthy	DP < 10 OF	Sucrose	10	35	No	No	Yes	Yes	Yes
Chen, 2000 [69]	Sequential	5	Constipated	DP < 10 scFOS	None	10	30	Yes	No	Yes	Yes	No
Den Hond, 2000 [70]	Cross-over	6	Constipated	DP ≥ 10	Sucrose	15	14	Yes	Yes *	Yes	Yes	Yes
Brighenti, 1999 [71]	Sequential	12	Healthy	DP < 10 OF	None	9	28	Yes	No	Yes	Yes	No
Tominaga, 1999 [72]	Parallel NB	34	Healthy	DP < 10 scFOS	Placebo	3	14	Yes	Yes	No	No	No ^f^
Van Dokkum, 1999 [73]	Cross-over	12	Healthy	DP < 10 OF & DP ≥ 10	Placebo	15	21	No	No	Yes	Yes	Yes
Sobotka, 1997 [74]	Sequential	9	Other	DP ≥ 10	None	30	7	Yes	Yes	No	No	No
Alles, 1996 [75]	Cross-over NB	24	Healthy	DP < 10 OF	Glucose	5 & 15	7	Yes	Yes	Yes *	Yes	Yes
Bouhnik, 1996 [76]	Parallel	20	Healthy	DP < 10 scFOS	Saccharose	12.5	12	No	No	No	Yes	Yes
Molis, 1996 [77]	Cross-over NB	6	Healthy	DP < 10 scFOS	30:70 Malto/sucrose	20.1	11	No	No	No	Yes	No
Gibson, 1995 [78]	Sequential	12	Healthy	DP < 10 scFOS & DP < 10 OF	None	15	15	Yes	No	Yes	Yes	No

^1^ Parallel or cross-over corresponds to a parallel or cross-over double blinded placebo-controlled study design; Parallel NB corresponds to a parallel non-blinded placebo- controlled study design; Cross-over NB corresponds to a cross-over non-blinded placebo-controlled study design; ^2^ scFOS corresponds to short-chain fructooligosaccharides from sucrose and OF to oligofructose. * No SD values. ** SD estimated on the basis of the lower and upper quartiles. ^a^ Patients with IBS based on pediatric Rome III criteria. ^b^ Patients with IBD diagnosed at least 6 months before enrolment, having experienced functional-like gastrointestinal symptoms that met Rome III criteria for IBS, functional bloating, or functionaldiarrhoea, and currently in remission, ^c^ Patients with active Crohn’s disease. ^d^ Stool frequency was measured but not reported in the publication. ^e^ Measurements described but no data reported on stool frequency and stool consistency in the publication. ^f^ Study individuals used as their own controls.

**Table 2:** Effect of β-fructans on stool consistency, fecal dry, and wet weights.

	All β-Fructans	Long-Chain β-Fructans (DP ≥ 10)	Short-Chain β-Fructans (DP < 10)
**Stool consistency** (Bristol stool scale score)			
*N* observations	6	1	5
Overall standardized mean difference (95% IC)	0.23 (0.05, 0.40)	NA	0.26 (0.08, 0.45)
*p*-value	0.01	NA	0.006
**Stool dry weight** (g/day)			
*N* observations	6	2	4
Overall standardized mean difference (95% IC)	0.18 (−0.05, 0.42)	0.31 (−0.35, 0.98)	0.17 (−0.08, 0.41)
*p*-value	0.12	0.35	0.19
**Stool wet weight** (g/day)			
*N* observations	12	3	9
Overall standardized mean difference (95% IC)	0.24 (0.06, 0.43)	0.29 (−0.22, 0.80)	0.24 (0.04, 0.43)
*p*-value	**0.008**	0.262	**0.016**

Bold type: significantly different *p* < 0.02.

## Supplementary Materials

The following are available online at http://www.mdpi.com/2072-6643/11/1/91/s1, Table S1: descriptive values of the various studies with respect to frequency of bowel movements taken into account in the present meta-analysis and meta-regression.
