# Supplementary material for: Effects of β-Fructans Fiber on Bowel Function: A Systematic Review and Meta-Analysis
Source: Nutrients. 2019 Jan 4;11(1):91. doi: 10.3390/nu11010091 (PMC6356805; doi:10.3390/nu11010091)
Supplement: Supplementary file 1 [file nutrients-11-00091-s001.pdf]

nutrients

MDPI

Supplemental Table 1. Descriptive values of the various studies with respect to frequency of bowel movements (number/day) taken into account in the present meta-analysis and meta-regression.

References	Dose (g/d)	DP	β-fructans			Placebo			Interval (days)	Age (years)	BMI (kg/m²)
			Mean	SD	N	Mean	SD	N			
Alles, 1996 [75]	15	<10	1.2	0.5	24	1.2	0.5	24	7	22.4	21.7
Alles, 1996 [75]	5	<10	1.3	0.5	24	1.2	0.5	24	7	22.4	21.7
Benamouzig, 2018 [32]	5	<10	0.72	0.39	75	0.64	0.32	75	42	32.9	24.2
Bouhnik, 2004 [64]	10	>10	1.2	0.09	8	0.91	0.09	8	15	30	-
Bouhnik, 2004 [64]	10	<10	1.05	0.11	8	0.91	0.09	8	15	30	-
Buddington, 2017 [35]	15	<10	0.65	0.04	49	0.58	0.03	48	84	33.4	29.1
Chumpitazi, 2018 [36]	6.2	>10	1	0.6	23	0.8	0.5	23	3	12.4	-
Closa-Monasterolo, 2017 [37]	4	<10	0.66	0.36	11	0.85	0.4	11	42	3.9	-
Costabile, 2010 [53]	10	>10	1.34	0.64	32	1.45	0.57	32	42	-	-
Cox, 2017 [38]	12	<10	2.3	1.8	29	1.9	1.3	29	3	39	-
Cummings, 2001 [67]	10	<10	1.52	0.59	117	1.45	0.44	127	35	50	-
Dahl, 2014 [45]	15.3	<10	1.43	0.74	30	1.39	0.73	31	56	24.25	25.5
Dahl, 2014 [45]	15.6	<10	1.84	0.86	20	1.83	0.82	17	56	24.25	25.5
Den Hond, 2000 [70]	15	>10	0.93	0.35	6	0.57	0.14	6	7	28.5	20.5
François, 2014 [46]	30	<10	1.2	0.4	20	1.2	0.3	20	14	46.9	24.4
Geyer, 2008 [56]	6.4	<10	1.3	0.001	16	1.1	4.2	16	14	29.3	-
Kleessen, 2007 [59]	7.7	>10	1.57	0.643	15	1.42	0.57	15	14	23.5	22.9
Kleessen, 2007 [59]	7.7	>10	1.29	0.5	15	1.42	0.57	15	14	23.5	22.9

Kolida, 2007 [60]	5	<10	1.508	0.633	30	1.478	0.674	30	14	26.5	-
Kolida, 2007 [60]	8	<10	1.461	0.735	30	1.478	0.674	30	14	26.5	-
Micka, 2017 [40]	12	>10	0.57	0.21	22	0.5	0.29	22	28	46.9	24.7
Ramnani, 2015 [44]	5	-	1.4	0.5	38	1.3	0.4	38	42	35	24.1
Ramnani, 2010 [54]	5	>10	1.35	0.51	22	1.39	0.5	22	42	32.8	24.3
Ramnani, 2010 [54]	5	>10	1.28	0.42	22	1.39	0.5	22	42	32.8	24.3
Ripoll, 2010 [55]	5	>10	1.2	0.09	18	1.49	0.11	17	28	-	-
Scholtens, 2006 [61]	30	<10	1.5	0.2	12	1.2	0.2	12	14	21.4	-
Slavin, 2011 [51]	20	>10	1.4	0.24	12	1.11	0.37	12	21	38	-
Swanson, 2002 [66]	3	<10	1.41	0.69	15	1.28	0.6	15	28	25.1	-
Wang, 2013 [49]	10	<10	0.75	0.3	50	0.41	0.05	50	10	56.1	-
Whelan, 2005 [63]	9.5	<10	0.9	0.3	10	0.6	0.2	10	14	27.5	25
Gendre, 2018 [33]	5	<10	0.56	0.27	93	0.49	0.26	94	42	38.9	24.1

- : information not provided.
